# Genetic Algorithm and Graph Theory Based Matrix Factorization Method for Online Friend Recommendation

**DOI:** 10.1155/2014/162148

**Published:** 2014-03-16

**Authors:** Qu Li, Min Yao, Jianhua Yang, Ning Xu

**Affiliations:** College of Computer Science, Zhejiang University, Hangzhou 310027, China

## Abstract

Online friend recommendation is a fast developing topic in web mining. In this paper, we used SVD matrix factorization to model user and item feature vector and used stochastic gradient descent to amend parameter and improve accuracy. To tackle cold start problem and data sparsity, we used KNN model to influence user feature vector. At the same time, we used graph theory to partition communities with fairly low time and space complexity. What is more, matrix factorization can combine online and offline recommendation. Experiments showed that the hybrid recommendation algorithm is able to recommend online friends with good accuracy.

## 1. Introduction

With the rapid development of social network, recommendation systems in various fields emerged. A good recommendation system should combine various kinds of recommendation effects and guarantee diversity on the base of accuracy, so as to satisfy some unpopular tastes.

Genetic algorithm does not need any a priori information about the distribution of experiment data [1]. It is often used in multiobjective optimization, a complex problem optimization field. It merges the information of individuals in the population, selects individual with high fitness, and finds the global optima by global search in relatively short time [2]. In real world social network, collective identification can emerge from strong connected, high density, and relatively isolated networks [3]. Each node in the network prefers to develop into highly integrated network group, that is, social circle. However, when the data is mass and sparse, time and space complexity will surge. Matrix factorization thus becomes an important dimension reduction technique.

## 2. Matrix Factorization

### 2.1. Matrix Factorization Mechanism

The basic idea of matrix factorization is to map raw “user-item” rating matrix into a common low-dimensional space. Item can be user or some kind of products specific to different applications. The dimension of low space is set to *k*, and the raw matrix is approximated by inner production of two low-dimensional matrices *U* = [*U*_1_, *U*_2_,…, *U*_*k*_] ∈ *R*(*N*∗*K*) and *I* = [*I*_1_, *I*_2_,…, *I*_*k*_] ∈ *R*(*M*∗*K*). Singular value decomposition (SVD) is the most famous matrix factorization method. When computing similarity, if there is no rating between user and item, it uses character vector and user item bias derived by matrix factorization, uses ${\hat{r}}_{ui}=q_{i}^{T}p_{u}$ to compose predicted ratings, that is, and uses SVD to fill in the sparse matrix [4].

### 2.2. Defects of Matrix Factorization

Classic matrix factorization method to some extend optimizes high-dimensional sparse matrix. However, neither user-based recommendation nor item-based recommendation algorithms considered the bias between the items itself and the group. In this paper, we proposed matrix factorization model with latent factor of user and item bias.

### 2.3. Improved Matrix Factorization

**Figure 1:**
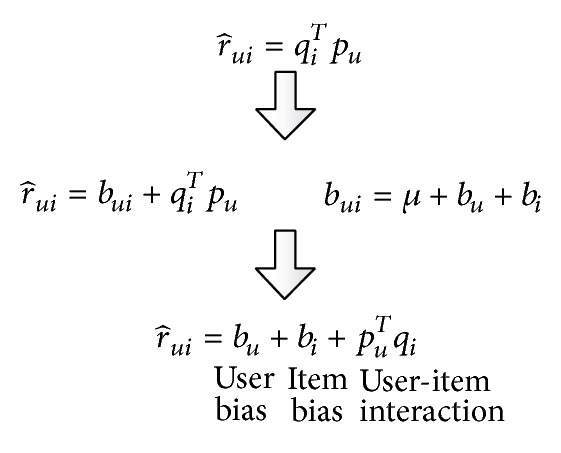
User rating prediction.

By adding variables such as user, item bias, and global factor into baseline prediction model, we get improved matrix factorization model. *U* is user collection; *I* is item collection. We use *u* and *i* to represent a user and an item, respectively; ${\hat{r}}_{ui}$ is *u*'s rating on item *i*. *R* is the raw user-item rating matrix; not null rating subset of (*u*, *i*) is *R* ^+^; null rating subset of (*u*, *i*) is *R* ^−^. According to SVD theory, rating matrix *R* can be factorized as two low-dimensional vector spaces. Thus, predicted rating of *u*'s rating on item *i* under baseline prediction model can be

(1) $$\begin{array}{c} {\hat{r}}_{ui}=f\left(b_{ui}+q_{i}^{T}p_{u}\right), \end{array}$$

where *f*(·) is a mapping function which can transfer the actual value of user rating into a specific range and *f* is predefined. *p*_*u*_, *q*_*i*_ represent the *d*-dimensional user feature vector and the item feature vector. Bias vector *b*_*ui*_ is defined as

(2) $$\begin{array}{c} b_{ui}=\mu +b_{u}+b_{i}, \end{array}$$

where *μ* is global mean rating and *b*_*u*_ and *b*_*i*_ represent the user bias and item bias, as shown in Figure 1.

This model can predict users' preference of items fairly accurate. This can also be used as user recommendation in social networks. It can discover user's preference besides their explicit tags. A small error between prediction rating and actual rating usually means a good prediction. In this section, we use regularized least squares as target function

(3) $$\begin{array}{c} \min\overset{}{\underset{(u,i)\in K}{\sum }}{\left(r_{ui}-{\hat{r}}_{ui}\right)}^{2}+\lambda_{1}{||p_{u}||}^{2}+\lambda_{2}{||q_{i}||}^{2}+\lambda_{3}b_{u}^{2}+\lambda_{4}{b_{i}}^{2}. \end{array}$$

In this equation, *λ*_1_, *λ*_2_, *λ*_3_, and *λ*_4_ are parameters to avoid overfitting. These parameters are decided by the number of data samples; the larger the data is, the smaller the parameters would be. *p*_*u*_, *q*_*i*_ can be solved with least squares or stochastic gradient descent (SGD) method. The SGD updates the parameters based on the following equations:

(4) bu⟵bu+η(eui−λ1bu),bi⟵bi+η(eui−λ2bi),qi⟵qi+η(eui−λ3qi),pu⟵pu+η(eui−λ4pu),

where parameter *η* indicates the learning rate; *λ*_1_, *λ*_2_, *λ*_3_, and *λ*_4_ are parameters which define the strength of regularization; the complexity of each iteration is a linear function of the number of ratings; *e*_*ui*_ is the error of each iteration; *b*_*u*_, *b*_*i*_, *p*_*u*_, and *q*_*i*_ are regulated variables.

## 3. Graph Based Social Network Construction

### 3.1. Community Detection in Social Network

Many complex network benchmark problems were generated by real world social networks but remain of high resemblance to real networks. The strength of links between nodes represents similarity of users. Similarity can be calculated with users' coratings, common friends, tags, and keywords. When the similarity of two users is larger than a predetermined threshold, the two users are considered to be in the same community. Where the two users are bilateral followers, the similarity is set to 1. Generally speaking, will not be many high density nodes in a network, while users in social network tend to form network with high density; the highly resembled users are more likely to be in one community. Users' interest, following relationship, can exceed similarity threshold when they are in the same community.

### 3.2. Graph Constrain Based Matrix Factorization Model

When new users and items are added into the matrix, matrix is getting sparser and the computation load rises accordingly. The prediction accuracy of the matrix degenerates or even fails to converge. In this paper, we added relation between user and item into the baseline prediction model to perform community detection. We use social relation between users and similarity on multidimensional feature space to generate optimal community division and find top *N* similar users of target user. Then we predict the target user's acceptance according to the nearest neighbor users' acceptance to item being recommended.

### 3.3. Maximum Modularity Based Partition Model

In [5], the authors first constructed an unweighted directed network *G*, where *N* is the node set, *E* is the edge set, deg(*n*) is the degree of node, and *m* is the size of edges in the network. Modularity uses the contribution of each node as its local variable. It can be randomly partitioned; then it uses greedy strategy to adjust local value to get maximum modularity. The concept of modularity is defined as

(5) $$\begin{array}{c} Q\left(c\right)=\underset{c\in C}{\sum }\left[\frac{\left|E\left(c\right)\right|}{m}-{\left(\frac{\sum_{n\in N}\deg\left(n\right)}{2\;m}\right)}^{2}\right]. \end{array}$$

*E*(*c*) is the inner edge set of community *c*; a smaller second part of the equation can guarantee the network division to be more refined, thus leading to a more compact community. In this paper, we use maximum modularity theory to find communities.

### 3.4. Genetic Algorithm and Multifeature Fusion Based Community Partition

Many genetic algorithms based methods have been proposed for community detection [6]. GA does not need to know the number of communities; only basic evolutionary parameters need to be defined. However, results show that, in large scale network, direct optimum search method does not perform well on communities of various sizes. Approximation of modularity does not reflect actual community of community structure. However, by combining nodes mapping to multidimensional matrix feature vector, it is possible to select the communities based on globally optimal modularity [3].

**Figure 2:**
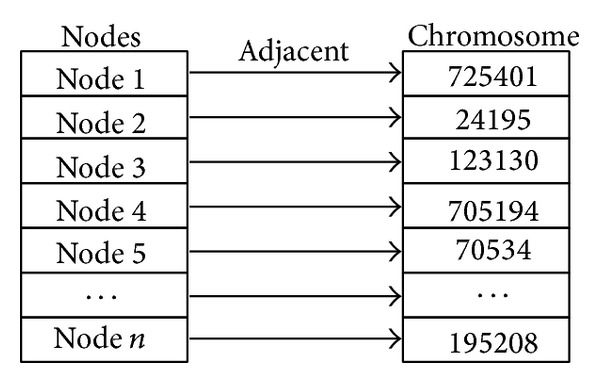
Node chromosome encoding.

In this paper, we combine the theory of hybrid genetic algorithm and matrix factorization to search for optimal modularity. Modularity is the fitness of individual. We use decimal number coding for chromosome. Each chromosome is composed of index of adjacent nodes. This kind of encoding method can avoid unviable solutions. For example, *g*_1_, *g*_2_,…, *g*_*n*_ is a chromosome composed of *n* genes. *g*_*i*_ is the index of an adjacent node of node *i*. For example, in Figure 2, gene 1 is 725401; it means node 725401 is adjacent to node 1.

Each gene in the chromosome is a randomly generated index in the range of [0, *n*]. In the iterations, the similarity between gene *g*_*i*_ and user *i* is calculated; if the similarity is larger than a predefined threshold, then the index in gene *g*_*i*_ and user *i* will be in the same community. Otherwise, two nodes are assigned into two communities. In the end, decoding process partitions the corresponding nodes of the same gene into multiple communities. Large modularity means a good partition of community division, and vice versa. *Q* often has a value in the range of [0.3, 0.7]. If there is no edge linking to node *i*, gene *g*_*i*_ will be assigned as the adjacent node of node *i*, assigning node *i* into the community with most users. Experiment shows that this kind of amend can effectively partition result.

### 3.5. User KNN and Matrix Factorization Based Model

To further improve the prediction model, we use *k* to represent the nearest neighbor of user *u*. *N* is the number of neighbors, sim(*u*, *k*) is the similarity between *u* and *k*, and *r*_*ki*_ is the predicted *k*'s rating on item *i*. *r*_*ui*_, which means *u*'s rating on item *i*, can be derived from the *N* nearest neighbors *k*'s rating on item *i*:

(6) $$\begin{array}{c} r_{ui}=\frac{\sum_{k=1}^{N}\text{sim}\left(u,k\right)\cdot r_{ki}}{\sum_{k=1}^{N}\text{sim}\left(u,k\right)}. \end{array}$$

The algorithm is roughly as follows.

 Input: user-item recommendation history, SNS relation.
 Output: friend recommendation list for target user.
  1. Use improved SVD to derive user, item feature vector, and bias.
  2. Combine multidimensional feature vector and users' explicit/implicit factors. Perform user community partition based on genetic algorithm.
  3. Search user's nearest neighbor on the base of community partition and multidimensional feature vector.
  4. Return to friend recommendation list for target user. Rank the list by acceptance rate.

## 4. Experiment Result

### 4.1. Dataset Preprocessing

We use the dataset of KDD Cup 2012 competition track1 provided by Tencent Weibo for experiment [5]. This dataset includes user attributes, SNS relations, user action, item attributes, tag, and other social network information. There are 2.32 million users, 6,000 items, 3 million user action data, and 300 million recommendation records. With respect to hardware limitation, we only choose data related to some relatively active users. We choose 2,000 users, 721 items (recommended users), 16,234 follow relations, and 20,000 recommendations. We divide the recommendation records into training set with 16,000 records and testing set with 4,000 records.

Only dataset with obvious network community structure is valuable for partition. In order to select sample with obvious community structure, we first use Gephi to analyze SNS structure. The dataset is finally divided into 9 communities with average network degree of 7.43 and modularity of 0.34, which indicate that there is obvious community structure in the selected dataset. There are many ways to select active users. In this paper, we use the rule to delete inactive users: number  microblog = 0, number  friends = 0, number  followers = 0, and number  microblog = number  repost. After the deletion, the rule to select active user is number  followers > 300 and number  friends > 0. Finally we randomly choose 2000 users, 721 items (recommended users), and related interacting data.

### 4.2. Algorithm Evaluation

In this paper, we use RMSE as error measure of predicted ratings. It is commonly believed that RMSE enforced penalty on inaccurately rated items; thus it is more focusing on recommendation accuracy. The raw rating set is records; let records $\left[i]=[u,i,r_{ui},{\hat{r}}_{ui}\right]$, where *r*_*ui*_ means *u*'s rating on item *i*. ${\hat{r}}_{ui}$ means the predicted rating; *k* is the length of records; the RMSE is described in the following equation:

(7) $$\begin{array}{c} \text{RMSE}=\sqrt{\frac{\sum_{(u,i)\in \text{records}}^{}{\left(r_{ui}-{\hat{r}}_{ui}\right)}^{2}}{k}}. \end{array}$$

In order to thoroughly investigate the performance of proposed algorithms, we use different standards at different stages of the experiment. RMSE is used for user feature vector computation. *Q* is used in community partition. Top *N* items are returned for accuracy evaluation. The accuracy of recommendation is measured by the RMSE of testing set [7, 8].

### 4.3. Experiment Results

**Figure 3:**
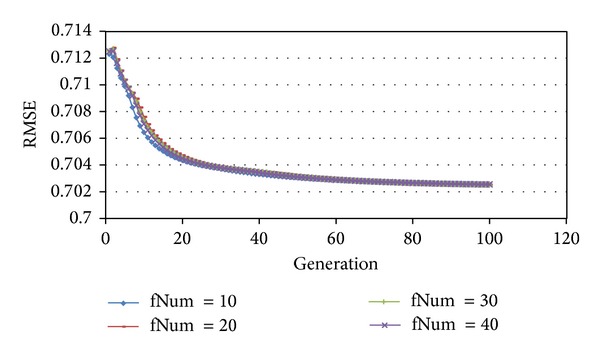
The effects of feature dimension on RMSE.

For matrix factorization, a higher feature dimension means a more refined partition. However, the higher feature dimension is, the slower the computation would be. Because of the randomness of genetic algorithms, we need to get best parameters by running the experiment several times with different settings. We use 10, 20, 30, and 40 for fNum, the feature dimension. The RMSE is as shown in Figure 3.

**Figure 4:**
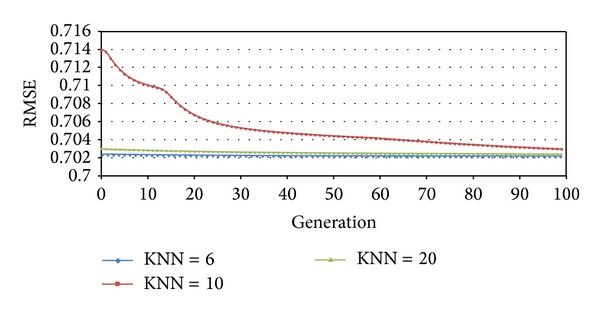
RMSEs of different KNNs.

From experiment results, we can see that high dimension does not necessarily lead to high accuracy. After 60 generations, fNum = 20 performs the best. Before that, there is no significant difference between different settings, so we choose fNum = 20. Also, from below Figure 4, it is easy to see when KNN is set to 6, algorithm performs better than 10 and 20. In this paper, for SVD + Bui + AVG + KNN model, KNN = 6, 10, 20, where Bui is the bias of user *u* against item *i*. The RMSEs change as shown inFigure 4.

**Figure 5:**
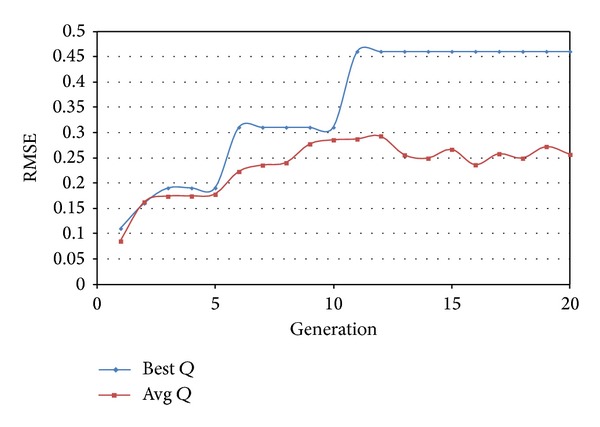
Result of community partition.

We compared the recommendation result of traditional SVD with our hybrid recommendation algorithm. Below is the result of algorithm with refine factor. We used parameters as follows: learning rate *η* = 0.01, regularization coefficient = 100, iteration number = 100, and number of KNN = 10. In the process of community partition, the parameters used are crossover probability *P*_*c*_ = 0.8, mutation probability *P*_*m*_ = 0.001, population size Popsize = 50, and max generation Maxgeneration = 20. The result of RMSE and community partition is shown in Figure 5.

**Figure 6:**
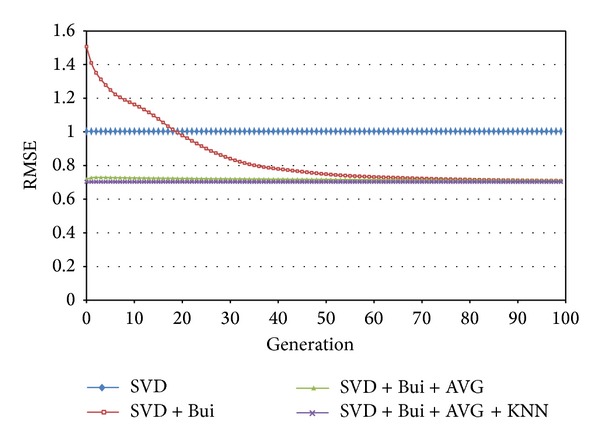
RMSE of proposed algorithm and basic SVD.

In Figure 6, we can see that by adding user and item bias (Bui), results are greatly improved. In order to get better convergence and accuracy, results with AVG and KNN are also compare with basic SVD. The RMSEs of four models are given in Table 1.

## 5. Conclusion

In this paper, we proposed a hybrid genetic algorithm and graph theory based online recommendation algorithm. Hybrid genetic algorithm is used to multiobjective combinatorial problem. We also used graph theory to partition the community. When target user prediction is performed, there is no need to search the whole user space, thus reducing the time and space complexity and solved the problem of cold start and data sparsity.

Traditional recommendation tends to overlook users and items' feature vector. In this paper, we used SVD matrix factorization to model user and item feature vector and used stochastic gradient descent to amend parameter and improve accuracy. To tackle cold start problem and data sparsity, we used KNN model to influence user feature vector. At the same time, we used graph theory to partition communities with fairly low time and space complexity. What is more, matrix factorization can combine online and offline recommendation.

We used the dataset of KDD Cup 2012 competition track1 provided by Tencent Weibo. Experiments showed that the hybrid recommendation algorithm is able to recommend online friends with good accuracy.

## Figures and Tables

**Table 1:** RMSE comparison of different models.

Number	Algorithm	RMSE
1	SVD	1
2	SVD + Bui	0.709202
3	SVD + Bui + AVG	0.703258
4	SVD + Bui + AVG + KNN	0.702454
